# Supplementary material for: Neutrophil-to-Lymphocyte, Monocyte-to-Lymphocyte, Platelet-to-Lymphocyte Ratio and Systemic Immune-Inflammatory Index in Different States of Bipolar Disorder
Source: Brain Sci. 2022 Aug 4;12(8):1034. doi: 10.3390/brainsci12081034 (PMC9405738; doi:10.3390/brainsci12081034)
Supplement: Supplementary file 1 [file brainsci-12-01034-s001.zip › brainsci-1834210-supplementary.pdf]

Supplementary Materials

Table S1- Supplementary materials Prognostic accuracy of white blood cells, platelets and Inflammatory Ratios in patients (manic or depressive episode)

Prognostic marker	Cut-off	Sensitivity	Specificity	PPV	NPV	AUC	95% CI	Sig.
**Neutrophil**	4.21	75.4%	36.9%	5.9%	96.6%	0.500	0.414-0.586	0.998
**Monocyte**	0.29	99.9%	0.9%	5.0%	99.4%	0.431	0.345-0.518	0.518
**Lymphocyte**	1.31	95.7%	7.2%	5.2%	97.0%	0.441	0.356-0.526	0.171
**Platelet**	233.5	62.5%	43.2%	5.5%	95.6%	0.470	0.383-0.557	0.497
**NLR**	1.43	92.8%	20.7%	5.8%	98.2%	0.543	0.457-0.628	0.326
**MLR**	0.16	97.1%	89.2%	32.1%	99.8%	0.480	0.394-0.566	0.644
**PLR**	140.7	30.4%	79.3%	7.2%	95.6%	0.525	0.438-0.612	0.577
**SII index**	634.9	40.6%	69.7%	6.6%	95.7%	0.524	0.437-0.612	0.583

PPV: Positive predictive value; NPV: Negative predictive value; AUC: area under the curve; CI: Confidence interval

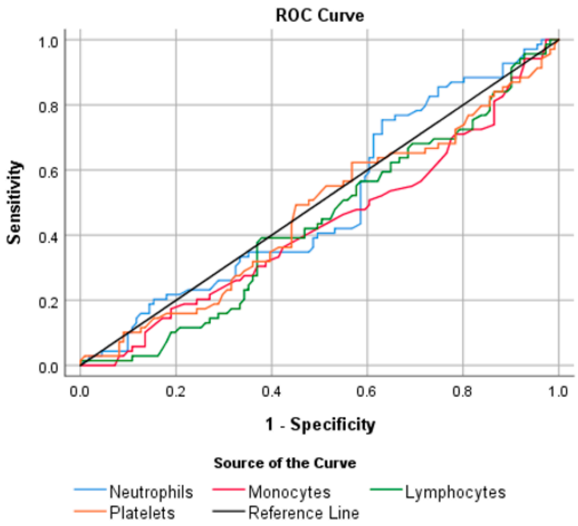

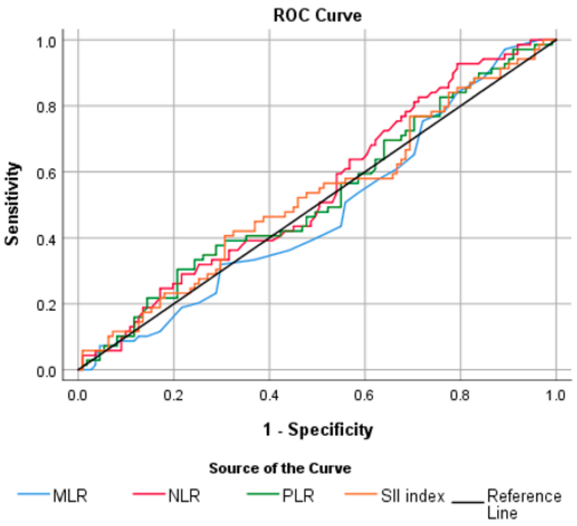

Figure S1- Supplementary materials. ROC curves of the of white blood cells, platelets (A) and Inflammatory Ratios (B) in patients (manic or depressive episode)
